# Large scale self-assembly of plasmonic nanoparticles on deformed graphene templates

**DOI:** 10.1038/s41598-021-91697-z

**Published:** 2021-06-10

**Authors:** Matthew T. Gole, Zhewen Yin, Michael Cai Wang, Wayne Lin, Ziran Zhou, Juyoung Leem, Satoshi Takekuma, Catherine J. Murphy, SungWoo Nam

**Affiliations:** 1 Department of Chemistry, University of Illinois at Urbana-Champaign, Urbana, IL USA; 2 Department of Mechanical Engineering, University of South Florida, Tampa, FL USA; 3 Department of Mechanical Science and Engineering, University of Illinois at Urbana-Champaign, Urbana, IL USA; 4 Department of Medical Engineering, University of South Florida, Tampa, FL USA; 5 Department of Materials Science and Engineering, University of Illinois at Urbana-Champaign, Urbana, IL USA

**Keywords:** Nanoscale materials, Graphene, Nanoscale materials, Materials chemistry, Chemistry, Nanoscience and technology

## Abstract

Hierarchical heterostructures of two-dimensional (2D) nanomaterials are versatile platforms for nanoscale optoelectronics. Further coupling of these 2D materials with plasmonic nanostructures, especially in non-close-packed morphologies, imparts new metastructural properties such as increased photosensitivity as well as spectral selectivity and range. However, the integration of plasmonic nanoparticles with 2D materials has largely been limited to lithographic patterning and/or undefined deposition of metallic structures. Here we show that colloidally synthesized zero-dimensional (0D) gold nanoparticles of various sizes can be deterministically self-assembled in highly-ordered, anisotropic, non-close-packed, multi-scale morphologies with templates designed from instability-driven, deformed 2D nanomaterials. The anisotropic plasmonic coupling of the particle arrays exhibits emergent polarization-dependent absorbance in the visible to near-IR regions. Additionally, controllable metasurface arrays of nanoparticles by functionalization with varying polymer brushes modulate the plasmonic coupling between polarization dependent and independent assemblies. This self-assembly method shows potential for bottom-up nanomanufacturing of diverse optoelectronic components and can potentially be adapted to a wide array of nanoscale 0D, 1D, and 2D materials.

## Introduction

Graphene-based devices have emerged as potentially versatile components for nanoscale optoelectronics applications^1^. Graphene’s zero-gap band structure with its unique linear energy–momentum relationship near the Dirac points yields a 2.3% wavelength-independent absorbance throughout the visible and near-IR ranges^2,3^. Such unique optoelectronic properties, especially for an atomically-thin material, have led to intensive research in graphene photonics including surface-enhanced Raman spectroscopy^4,5^, photocatalysis^6^, and photodetection^7,8^. Graphene-based devices such as photodetectors are superlative for their versatility across a wide range of wavelengths^9^. However, spectral and polarization specificity/sensitivity are often desired for applications in high speed and compact photonic devices^10,11^. As monolayer graphene does not inherently possess these properties, there is interest in hybrid photonic systems that introduce multifaceted optical responses to graphene-based metasurfaces.

One way to increase properties such as efficiency and spectral selectivity of graphene-based metasurfaces is through coupling of the incident excitation with surface plasmon resonance (SPR) structures^12–18^. Gold nanostructure integration is a common approach for plasmonically controlling the optical response of host materials. Plasmonic nanostructures form enhanced, highly-localized electromagnetic fields under illumination and have been shown to enhance the photoabsorption of graphene to achieve 20 times higher photoresponsivity^12^. The geometry of the gold structures can determine the polarization selectivity of metasurfaces^12,15^, with the strongest enhancement occurring selectively at wavelengths matching with the SPR frequency of the nanostructures^17^.

To date, plasmonic nanostructure integration on graphene and other 2D materials has largely been achieved through pattern definition (e.g. lithography) and metallization (e.g. metal deposition/evaporation). While precise, these conventional methods have limitations in scalability and geometric control, respectively. In contrast, plasmonic gold nanoparticles (AuNPs) can be colloidally synthesized with high yield and with a very large diversity in morphology (shape, size, aspect ratio). However, the potential of colloidal nanoparticles for solid state devices has been limited due to their tendency to form arbitrary, non-uniform, and/or close-packed aggregates upon assembly onto surfaces^19^. This results in poor control over the nanostructure geometry, with concomitant poor performance of the resulting metastructure and devices.

One way that colloidal AuNPs have been integrated with substrates is through templated assembly, which allows for large-scale bottom-up geometric control of nanoparticle arrays^20–24^. Controllable assembly depends on architectured templates with nanoscale features to confine particles of varying shapes and sizes as well as control over the surface coverage and interparticle interactions. Nanoscale trough-like features used for single particle alignment are achievable in 2D materials such as graphene through mechanical deformation. Such alteration of the surface topography of graphene through mechanical deformations has previously been studied to enhance its mechanical^25,26^ and optoelectronic^27^ properties. Buckle delamination on stretchable substrates results in uniaxially aligned crumples with a periodicity on the order of tens of nanometers^28, 29^. Additionally, graphene can be conformally wrinkled on deformed elastomers with precise control over the wrinkle morphology, wavelength, and amplitude^30,31^. Mechanically deformed elastomer templates (purely polymeric without 2D materials) have previously been used to assemble colloidal AuNPs^22,32^. To date, templated assembly of nanoparticles on deformed or architectured 2D materials has not yet been explored.

Here we report a bottom-up approach for 2D/0D heterostructures with tunable anisotropic optical properties by using graphene templates which were fabricated via strain induced mechanical surface instabilities to deterministically assemble AuNPs. By controlling processing parameters, we designed deformed graphene templates with uniaxial buckle delaminated wrinkles with a periodicity of a few dozen nanometers, as well as aligned sinusoidal wrinkles spanning up to several hundred nanometers to match the dimensionalities of monodisperse AuNPs. The surface coating of the AuNPs was optimized for colloidal stability and uniformity under the deposition and assembly conditions and could be further altered to increase the steric repulsion between AuNPs and to enable further tunability to the metasurface optical properties. This novel method for self-assembly of nanomaterial heterostructures allows independent control over the various processing factors for each nanomaterial and resulting metastructure properties.

## Results and discussion

### Fabrication of buckle delaminated graphene templates and AuNP surface functionalization

**Figure 1:**
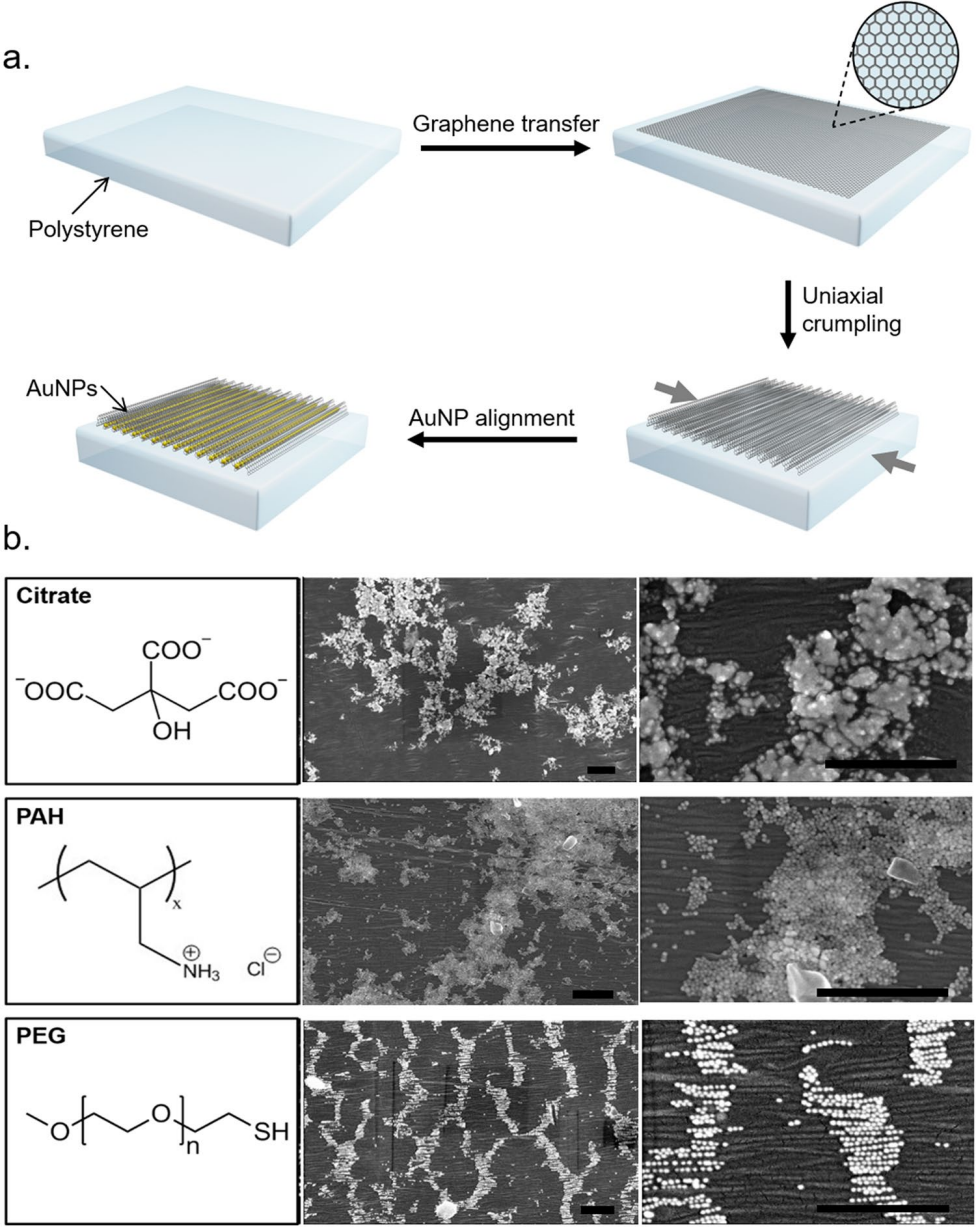
Schematic of graphene buckle delamination and gold nanoparticle assembly, and SEM images of gold nanoparticles with varying surface chemistry deposited onto buckle delaminated graphene. Schematic of graphene buckle delamination and gold nanoparticle assembly (**a**). SEM images of 20 nm AuNPs with citrate, poly(allylamine hydrochloride), and poly(ethylene glycol) surface coatings, after deposition and drying on graphene substrates (**b**). All scale bars 500 nm. Schematic was created with Autodesk 3ds Max software (version 2018, http://www.autodesk.com).

Fabrication of buckle delaminated graphene substrates was adapted from previous work^28^, and is outlined in Fig. 1a. Graphene with a gold handle layer was transferred to precleaned, shape-memory polystyrene sheets by standard wet transfer techniques. The gold handle layer was employed to avoid organic residues that are unavoidable with commonly used polymer handle layers.

20 nm citrate capped AuNPs were synthesized by the Turkevich method^33^. Several surface functionalizations were tested to optimize the AuNPs for alignment and resistance to aggregation. The as-synthesized citrate ligands on AuNPs served as a precursor for further particle functionalization, due to its high density of charge as well as potential for displacement for subsequent functionalization. The anionic charge was employed for the wrapping of the particles with positively charged poly(allylamine hydrochloride) (PAH) polyelectrolyte. Thiolated poly(ethylene glycol) (PEG) was also used to functionalize AuNPs with hydrophilic, neutrally charged polymer brushes.

As shown in Fig. 1b, there is a large contrast in AuNP behavior for different surface functionalizations. Particles coated with the native citrate, as well as PAH, both experience aggregation upon drying on crumpled graphene surfaces. This was attributable to the increase in both the particle concentration and the ionic strength of the solution during evaporation of the carrier solvent^34^. Particles coated with PEG were found to align within the wrinkles and resist aggregation upon drying. The primarily steric stabilization of the PEG molecules also prevented aggregation that is prevalent for the particles stabilized electrostatically. PEG functionalization has the additional advantage of allowing for the removal of excess capping agents through repeated washing in water. Thorough washing of the particles was pivotal to prevent substrate fouling by organic residues which interfere with the uniformity of the self-assembly.

While graphene crumples resulted in aligned AuNPs due to geometric confinement, there were several factors that presented challenges for large-scale uniform assembly and tunability. The high aspect ratio of the crumples often leads to substrate dewetting during the AuNP assembly process, resulting in striped patterns due to stick–slip behavior as seen in Fig. 1b. Additionally, the dimensions of the crumple troughs, being determined by the mechanical properties of graphene, limited the range of tunability of the template for different size nanoparticles.

### Fabrication of conformally wrinkled graphene templates

To access a broader range of characteristic length scales and to assemble AuNPs of various sizes, we adopted a conformal uniaxial wrinkling approach, whereby the 2D material (e.g. graphene) conforms to the corrugations of the underlying polymeric substrate. Since this approach is mechanistic in nature (does not require chemical interaction between the nanoparticles and the 2D surface), the self-assembly process is material-agnostic and extendable to assembly onto arbitrary 2D materials and thin film templates.

**Figure 2:**
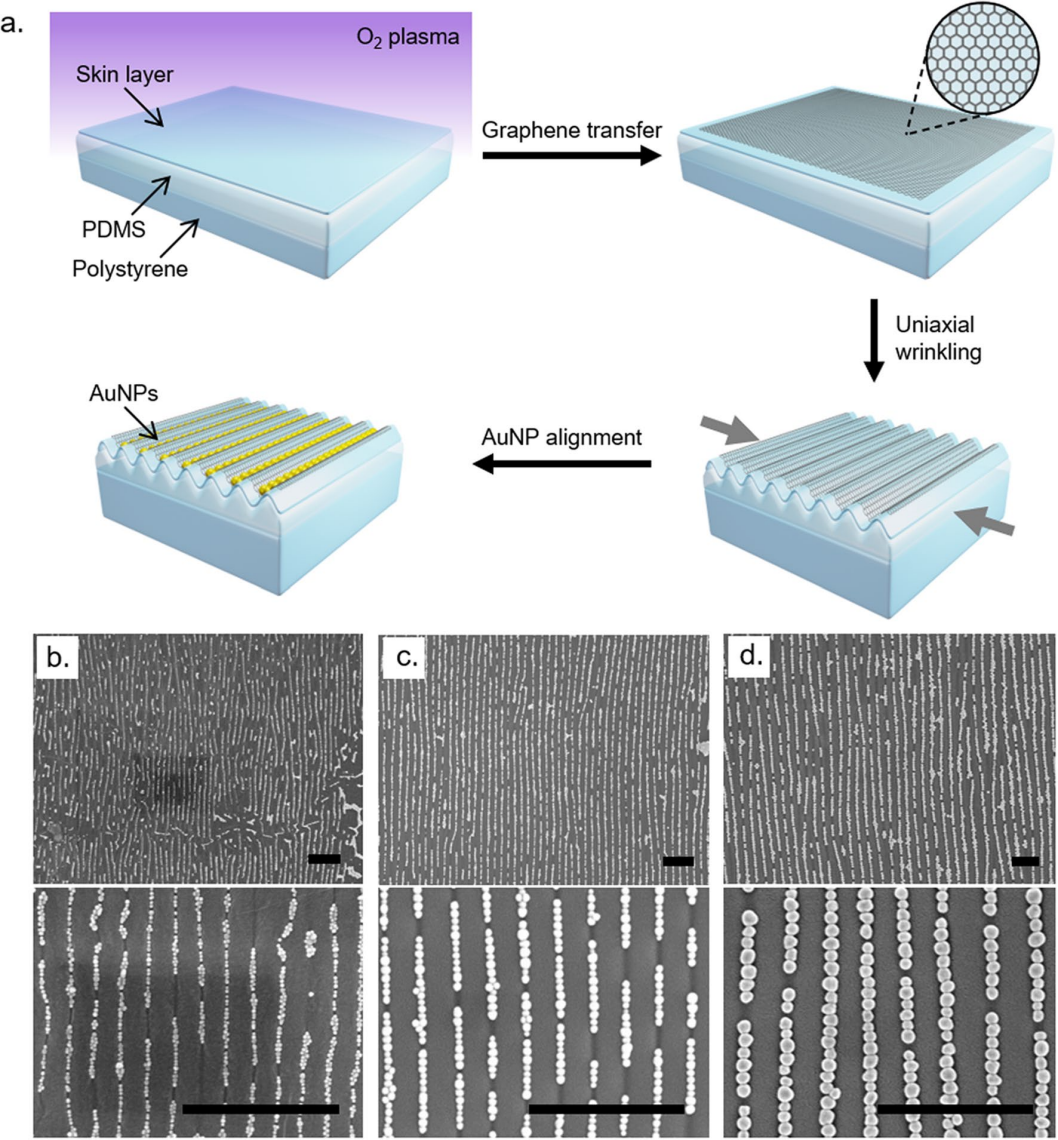
Schematic of conformal graphene wrinkling on PDMS and gold nanoparticle assembly, and SEM images of gold nanoparticles aligned on conformal wrinkled graphene. Schematic of wrinkled substrate assembly and AuNP deposition (**a**), SEM micrographs of 20 nm (**b**), 50 nm (**c**), and 80 nm (**d**) AuNPs on wrinkled graphene. All scale bars 1 μm. Schematic was created with Autodesk 3ds Max software (version 2018, http://www.autodesk.com).

Fabrication of the architectured 2D substrates is outlined in Fig. 2a. Beginning with cleaned, shape-memory polystyrene (PS) sheets that are surface treated to improve the wettability^35^ as well as the bonding to PDMS, a ~5 μm layer of PDMS elastomer was bonded to the PS by spin coating, followed by treatment with tens of seconds of O_2_ plasma at low power to create a rigid silica skin layer with a thickness of ~6–11 nm^36^. Tuning this silica layer thickness by increasing plasma time and power (dosage) allows for fine control of the final wrinkle dimensions due to the relative mismatch in plane-strain moduli between PDMS and plasma-induced silica skin layer^37^. Graphene is then transferred onto the silica/PDMS surface by standard wet transfer procedures using a thin film gold handle layer as mentioned previously. Conformal uniaxial wrinkling of the graphene was performed through the same shrinking procedure as for buckle delaminated graphene. This resulted in sinusoidal wrinkles with controlled wavelength and amplitude within which AuNPs could be self-assembled.

### Nano-capillary self-assembly of AuNPs

In addition to the 20 nm AuNPs employed for crumpled graphene, 50 and 80 nm citrate capped AuNPs were synthesized using a seed-mediated approach^38^. The AuNPs were self-assembled onto the graphene templates (Fig. 2b–d) by drop-casting the ethanolic colloidal AuNP solution onto a freshly made substrate of appropriate characteristic length scales (wrinkle wavelength and amplitude). A particle concentration of 4 nM was used for 20 nm AuNPs, and 0.5 nM for 50 and 80 nm AuNPs. The sample was then kept perfectly levelled and left to dry in a saturated ethanol environment. Care was taken to ensure the sample surface and surrounding environment were undisturbed to maintain uniform solution coverage on the template surface, thus preventing the meniscus from breaking and forming undesired coffee rings or local film dewetting during the deposition and drying process. AuNPs spontaneously aligned into the wrinkle valleys to maximize van der Waals forces between the particles and the sidewalls of the template^21^, and additionally experienced densification due to capillary forces between partially submerged particles^39,40^.

**Figure 3:**
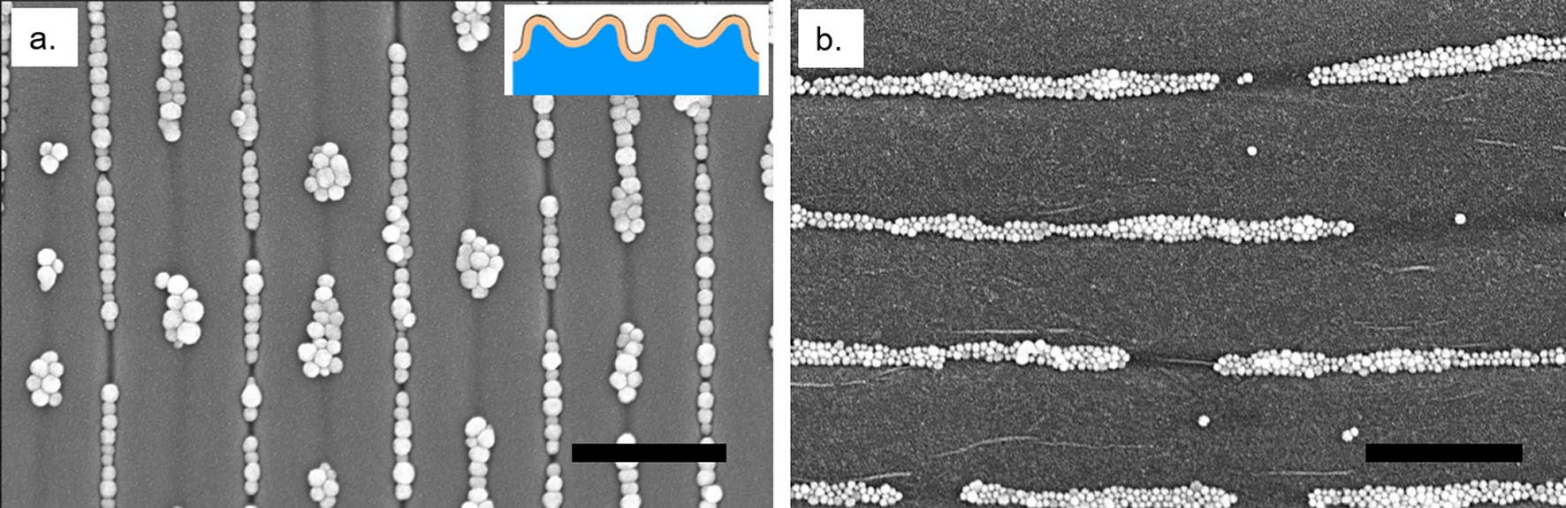
Effects of excessive strain and oversized wrinkles on nanoparticle alignment. Defects in AuNP alignment due to excessive strain causing period doubling (inset: a schematic of period doubled winkles) (**a**). Oversized wrinkles leading to overfilling of NPs (**b**). Scale bars 500 nm.

Wrinkle geometry was crucial for uniaxial single file assembly of AuNPs. Wrinkle wavelengths were tuned via O_2_ plasma treatment time to optimize alignment. An applied uniaxial strain of 9–11% was found to yield the most uniform wrinkles, with excessive strain resulting in topological defects such as period doubling and bifurcations (Fig. 3a), and too little strain causing the formation of creases that are too shallow and/or wide for single file particle assembly (Fig. 3b).

Transferring aligned plasmonic nanomaterials to other substrates may be desirable for some applications. While the PDMS substrate allows for more controllable templated assembly as compared to graphene crumples, the strong adhesion of graphene to the substrate limits the ability to transfer wrinkled graphene with aligned AuNPs to arbitrary substrates. We have previously reported some success with transferring of crumpled graphene to SiO_2_ substrate via solvent dissolution of the polystyrene substrate followed by wet transfer^28^. Crosslinked PDMS, however, is resistant to solvents and is thus much more challenging to isolate from the graphene.

### Optical properties of aligned AuNPs

A useful aspect of assembled AuNPs is the unique optical properties that emerge distinct from their intrinsic forms. When a plurality of noble metallic nanostructures are in close proximity, their individual modes can interact and form new hybrid modes with higher order interactions due to multiple scattering^41,42^. Single file alignment, in particular, gives rise to two orthogonal plasmonic modes with distinct resonance frequencies^22,43^. Such properties would potentially be effective for spectral and polarization selective devices.

**Figure 4:**
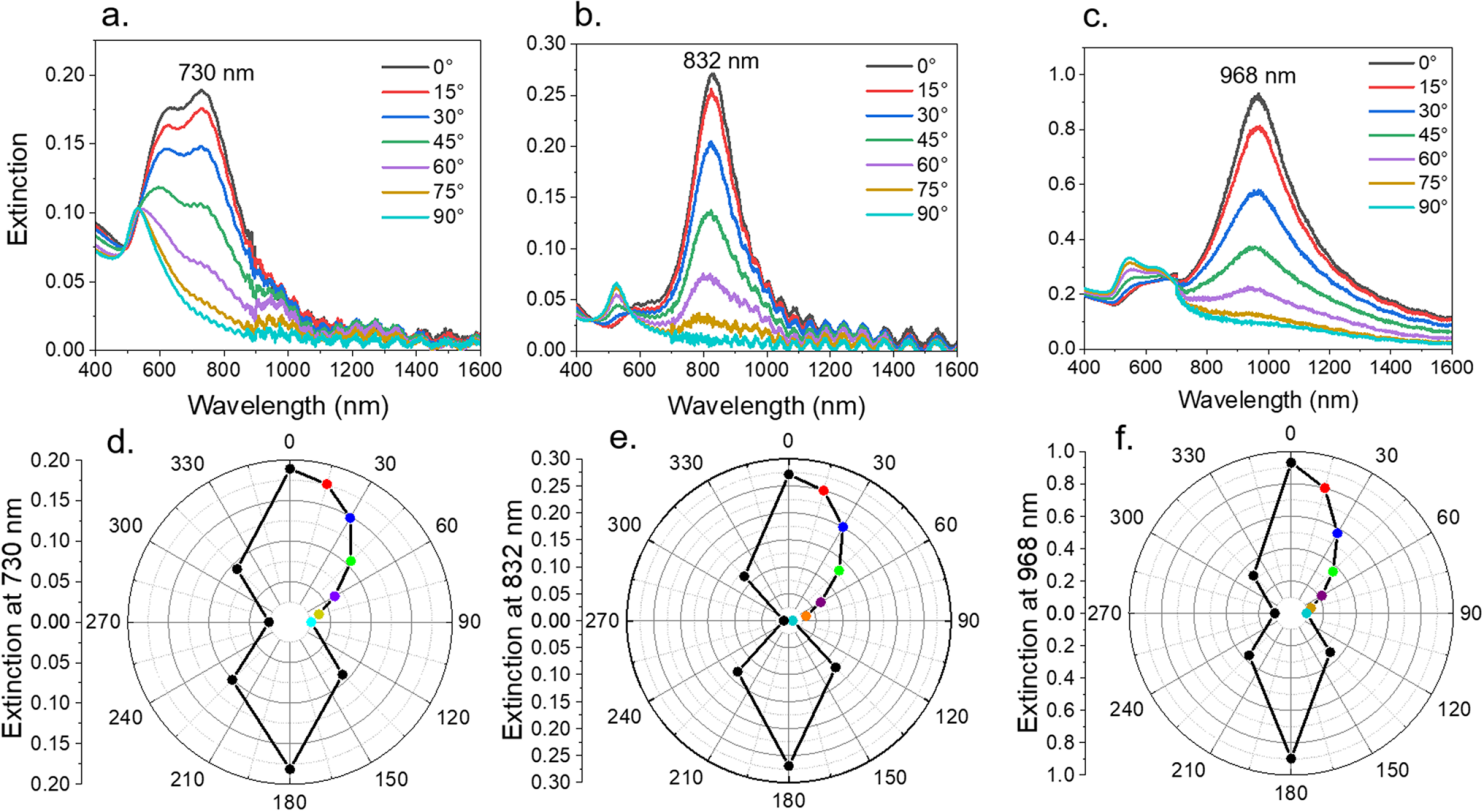
Polarized absorption spectra of aligned AuNPs. Polarized extinction spectra of 20 nm (**a**), 50 nm (**b**), and 80 nm (**c**) AuNPs on wrinkled graphene. Polar plots show the extinction values at λ_max_ at each polarization angle for 20 nm (**d**), 50 nm (**e**), and 80 nm (**f**) AuNPs.

To analyze the optical properties of the aligned AuNPs of different sizes, polarized UV–Vis measurements of the 2D substrates were taken through a Glan–Thompson polarizer (Fig. 4a–c). Measurements were made at 15° intervals from 0°–90°, then at 45° intervals for the remaining rotation. Spectra from 0°–90° are shown in Fig. 4a–c. When polarization is at 0° (parallel to the AuNP chains), a strong red-shift in the plasmonic resonance occurs which is a result of a decrease in the Coulombic restoring force due to plasmonic coupling^43^. The location of this peak varied with particle size, with larger particles being more red-shifted. The 20, 50, and 80 nm AuNPs coupled to give resonances at 730 nm, 832 nm, and 968 nm, respectively. The large shoulder on the blue side of the 20 nm AuNP spectrum is likely a result of defects causing a small amount of multiple filling^22^. This was not as pronounced for 50 and 80 nm AuNPs due to better uniformity of larger wrinkles. The periodic rippling in each spectrum was due to optical interference from the spin-coated PDMS layer. The polarization dependent properties are further illustrated through polar extinction plots (Fig. 4d–f). The longitudinal extinction for each particle size decreases with a cosine squared dependence, as predicted by Malus’ Law.

### Increasing spacing of AuNPs

**Figure 5:**
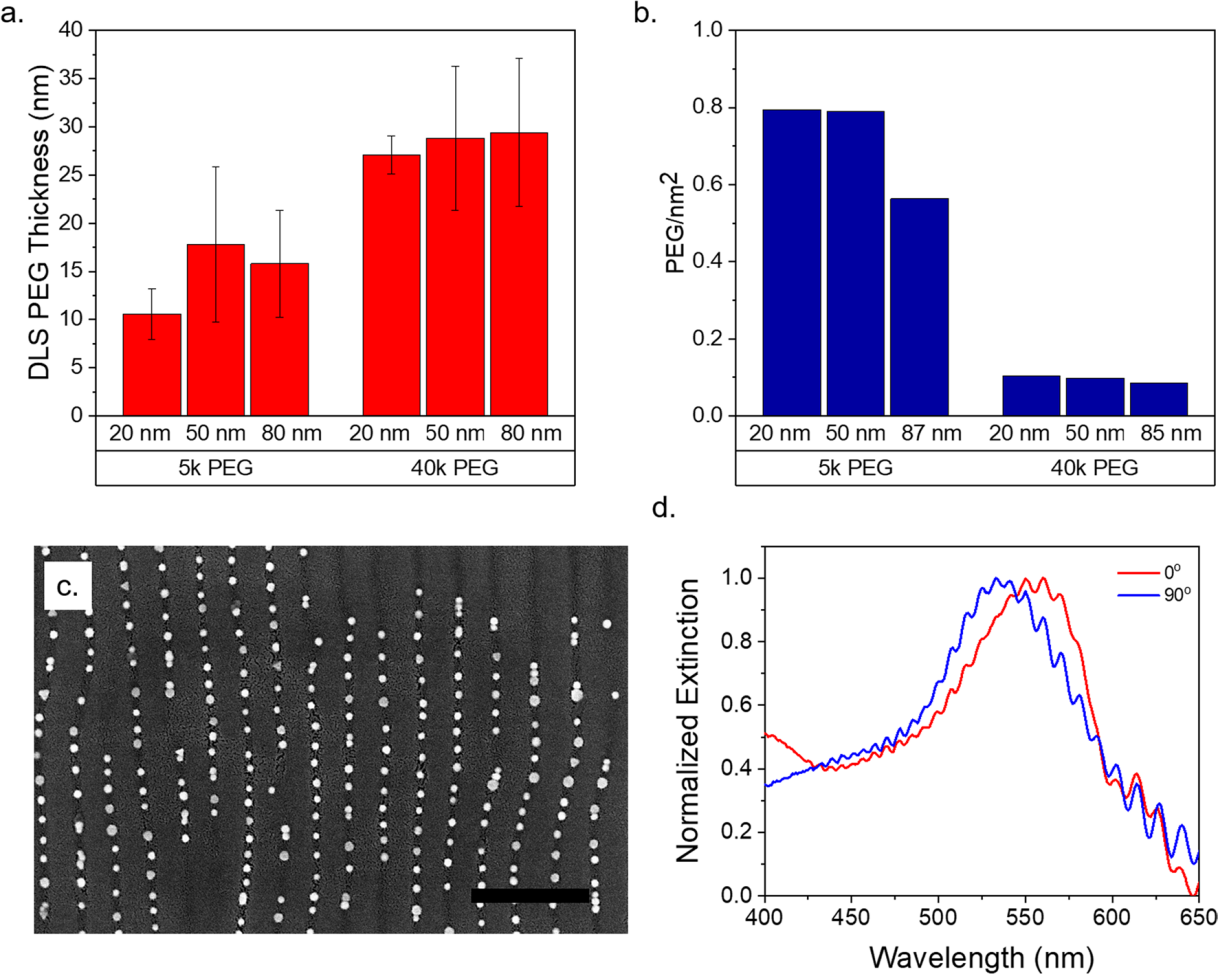
DLS and TGA measurements of PEG-coated AuNPs, SEM image of aligned 20 nm AuNPs on wrinkled graphene, and polarized UV–Vis spectra. PEG hydrodynamic thickness in ethanol on 20–80 nm AuNPs determined by DLS (**a**). PEG grafting density determined by TGA (**b**). SEM (**c**) and UV–Vis spectra (**d**) of 20 nm AuNPs capped with 40k PEG on graphene. Scale bar 500 nm. Error bars are the standard deviation of 3 replicates.

To further tune the optical properties of the aligned AuNPs we altered the interparticle spacing to modulate the amount of plasmonic coupling between particles^44,45^. Our strategy for this was to increase the length of the PEG polymer brushes grafted to the AuNPs to sterically hinder capillary densification. Dynamic light scattering (DLS) measurements (Fig. 5a) show the increase in PEG layer thickness by increasing PEG brush length. The scaling of size increase is less than N^3/5^, where N is the number of PEG monomers, suggesting the polymer conformations are in the mushroom regime^46^. This is also caused by ethanol being a poorer solvent compared to water for PEG. Thermogravimetric analysis (TGA) of dried particle samples shows an inverse relation between PEG grafting density and particle diameter (Fig. 5b). This is attributable to fewer low-coordinated surface gold sites present in larger nanoparticles, which are more favorable for citrate exchange by thiols^47^. Additionally, the grafting density decreases greatly with increasing PEG size due to steric effects.

The increase of PEG molecular weight from 5000 to 40,000 results in semiregular spacing of nanoparticles with a great reduction in plasmonic coupling and polarization dependence (Fig. 5c,d). The ~50 nm spacing is sufficient to eliminate all coupling^44^. The slight red-shift that does occur at 0° is likely a result of disperse particle dimers/trimers that could not be fully prevented, potentially due to local variations in PEG grafting density on the AuNP surface. The ability to independently control the polarization dependence by altering the colloidal building block functionalization broadens the potential applications for bottom-up 0D/2D metasurfaces.

In summary, we have demonstrated a bottom-up approach for the deterministic assembly of plasmonic nanoparticles on architectured 2D graphene substrates. Conformal wrinkling of graphene on PDMS substrates provides a deterministic and scalable template for the controlled self-assembly of plasmonic AuNPs. Additionally, the ability to alter the nanoparticle spacing by modulating the molecular weight of grafted polymer brushes expands the potential for assembled AuNPs metasurfaces in general. Our work represents the first instance of large-scale geometric control of colloidally synthesized plasmonic nanoparticles integrated with 2D materials. The precise control over the alignment of nanoparticles provides a myriad of possibilities in designing metasurfaces for polarization-based electronics and sensors.

## Methods

### Materials

Monolayer graphene on copper foil was purchased from Grolltex (SC6 × 6). Polystyrene sheets were purchased from Scientific Explorer-Poof Slinky (item number 0SD300-10A). TFA gold etchant was purchased from Transene Company, Inc. Polydimethylsiloxane (Sylgard 184), hydrogen tetrachloroaurate (HAuCl_4_*3H_2_O) (> 99.9%), poly(allylamine hydrochloride) (molecular weight 50,000), isopropanol (ACS Plus grade), and 200 proof ethanol (HPLC grade) were purchased from Sigma-Aldrich. Sodium citrate dihydrate (lab grade) was purchased from Fischer Scientific. Methoxy-poly(ethylene glycol)-thiol (mPEG-SH, 5 kDa, 20 kDa, and 40 kDa) was purchased from Creative PEGWorks. Nanopure water (18 MΩ⋅m) was obtained from a Millipore system. All chemicals were used as received without further purification.

### Fabrication of wrinkled substrates

Polystyrene (PS) sheets were ultrasonicated in deionized water for 10 min then repeated in isopropanol. For buckle delaminated graphene, the graphene was transferred directly onto the polystyrene. For conformal graphene wrinkling, Sylgard 184 PDMS with a mixing ratio of 7:1 was first spin-coated at 6000 rpm for 90 s onto the polystyrene surface and then cured together at 80 °C for 12 h. To ensure bonding between the PDMS and PS, the PS sheet was treated by oxygen plasma at 250 W for 10 s in a Plasma Therm 700 before spin coating. Depending on the target silica thickness, the PDMS was then subjected to 10–60 s of oxygen plasma at 20 W. Graphene with a 50 nm evaporated gold handle layer was transferred to the substrates (PDMS or PS) by standard wet transfer procedures. The handle layer was etched with a 20 mg/mL KI, 5 mg/mL I_2_ solution in ethanol. To form aligned uniaxial wrinkles, the sample was clamped on opposite ends and heated at 102 °C for approximately 20 h until the target amount of compressive strain (30% for buckle delamination and 9–11% for conformal PDMS wrinkles) was applied.

### Synthesis and surface functionalization of gold nanoparticles

20 nm AuNPs were prepared by the Turkevich method^26^. 500 mL nanopure H_2_O and 1.25 mL 0.1 M HAuCl_4_ were brought to a boil while stirring at 600 rpm. 2.5 mL 20% sodium citrate solution (m/m) was added rapidly, and the reaction was stirred for 30 min at just below boiling, after which an additional 0.625 mL of sodium citrate solution was added. After 10 min, the particles were cooled to room temperature, centrifuged at 9000 rcf, and washed once with nanopure water.

AuNPs (50 and 80 nm) were prepared by the seed-mediated method of Chan and coworkers^38^. Gold seeds (~ 14 nm) were first prepared by the Turkevich method. For the growth solutions, 475 mL nanopure H_2_O and 1.27 mL 0.1 M HAuCl_4_ were combined in a flask followed by addition of gold seeds. The seed concentration used was 0.0619 nM for 50 nm AuNPs, and 0.0138 nM for 80 nm AuNPs. While stirring at 600 rpm, 110 μL of 10% sodium citrate (m/v) was added, followed by 5 mL 0.03 M hydroquinone. Particles were stirred 60 min more, then centrifuged at 3000 rcf and resuspended in nanopure water. AuNPs were characterized by TEM and UV–Vis spectroscopy.

For polyelectrolyte wrapping, 20 nm AuNPs were dispersed into 10 mL H_2_O at a concentration of 1 nM. 4 mL of 10 mg/mL poly(allylamine hydrochloride) (PAH) polymer and 2 mL of 0.01 M NaCl were mixed, then added to the AuNP solution. The mixture was incubated overnight, before centrifuging at 9000 rcf and discarding the supernatant containing excess polyelectrolyte. Particles were redispersed in 200 proof ethanol before deposition.

For mPEG-SH functionalization, aqueous AuNP solutions at a concentration of 1 nM were incubated in 0.2 mM mPEG-SH MW 5k or 1.6 mM mPEG-SH MW 40k overnight. Particles were transferred to clean centrifuge tubes and washed 6 × with nanopure water to remove all traces of organic residues. Particles were then pelleted once more and dispersed in 200 proof ethanol to the target concentration (4 nM for 20 nm AuNPs, 0.5 nM for 50 and 80 nm AuNPs). To determine the hydrodynamic thickness of PEG layers, dynamic light scattering (DLS) measurements of PEG-coated AuNPs in ethanol were taken with a Malvern Zetasizer Nano ZS and the difference compared to citrate capped AuNPs in water was calculated. PEG grafting density was determined through thermogravimetric analysis of lyophilized particle samples (~ 10 mg each) with a TA instruments QA TGA running in air.

### Nanoparticle deposition and assembly

The target substrate was first prewet with 20 μL of 200 proof ethanol followed by drop casting of 20 μL of ethanolic mPEG-SH functionalized AuNP solutions onto 1 cm^2^ wrinkled substrates. The deposition was performed to yield uniform wetting of the substrate with menisci pinned at the substrate edges. Samples were dried overnight in a saturated ethanolic atmosphere. Aligned AuNPs on graphene were characterized by SEM in a Hitachi S4800 Field Emission Scanning Electron Microscope (SEM) and Hitachi SU-70 SEM. Polarized UV–Vis measurements were performed with a Glan Thompson calcite polarizer in a Cary-5000 UV–Vis spectrometer.
